# Supplementary material for: Impact of the UK soft drinks industry levy on health and health inequalities in children and adolescents in England: An interrupted time series analysis and population health modelling study
Source: PLoS Med. 2024 Mar 28;21(3):e1004371. doi: 10.1371/journal.pmed.1004371 (PMC11008889; doi:10.1371/journal.pmed.1004371)
Supplement: S1 Text — (DOCX) [file pmed.1004371.s001.docx]

# Interrupted time series analysis

The model specification is :

*Yt = β_0_ + β_1_T + β_2_Z + β_3_Z****⋅****T + β_4_A + β_5_At + β_6_Z****⋅****A + β_7_Z****⋅****At + β_8_I + β_9_It + β_10_Z****⋅****I + β_11_Z****⋅****It + + β_12_ M + β_13_ D + β_14_ J + β_15_ Z****⋅****M + β_16_ Z****⋅****D + β_17_ Z****⋅****J*

| Yt | Mean purchased sugar in soft drinks per household/ week at week t (t=1,…,295) |
| --- | --- |
| T | Study Week number from the start of the study (eg: 1:295) |
| Z | Control category (toiletries) = 0, soft drinks = 1 |
| A | Pre-announcement = 0, post-announcement = 1 |
| A_t_ | Week number since announcement [0{106}, 1:189] |
| I | Pre-implementation = 0, post-implementation =1 |
| It | Week number since implementation [0{213}, 1:82] |
| M | Mean monthly temperature (degrees Celsius) |
| D | December = 1, all months excluding December =0 |
| J | January = 1, all months excluding January =0 |

And coefficients are interpreted as follows:

*β_0_* : Intercept for toiletries

*β_1_* : Trend change in purchasing of toiletries per week (pre announcement)

*β_2_* : Intercept for soft drinks

*β_3_* : Trend change in purchasing of soft drinks across time

*β_4_* : Step change in purchasing of toiletries (at announcement)

*β_5_* : Trend change in purchasing of toiletries per week (post announcement)

*β_6_* : Step change in purchasing of soft drinks (at announcement)

*β_7_* : Trend change in purchasing of soft drinks (post announcement)

*β_8_* : Step change in purchasing of toiletries (at implementation)

*β_9_* : Trend change in purchasing of toiletries per week (post implementation)

*β_10_* : Step change in drinks purchasing (at implementation)

*β_11_* : Trend change in drinks purchasing (post implementation)

*β_12_* : Seasonal variation in purchasing of toiletries with average monthly temperature

*β_13_* : Seasonal variation in purchasing of toiletries in December

*β_14_* : Seasonal variation in purchasing of toiletries in January

*β_15_* : Seasonal variation in purchasing of soft drinks with average monthly temperature

*β_16_* : Seasonal variation in purchasing of soft drinks in December

*β_17_* : Seasonal variation in purchasing of soft drinks in January
