# Supplementary material for: Impact of the UK soft drinks industry levy on health and health inequalities in children and adolescents in England: An interrupted time series analysis and population health modelling study
Source: PLoS Med. 2024 Mar 28;21(3):e1004371. doi: 10.1371/journal.pmed.1004371 (PMC11008889; doi:10.1371/journal.pmed.1004371)
Supplement: S2 Text — (DOCX) [file pmed.1004371.s002.docx]

# Lifetable model data inputs

# Population

We modelled outcomes for the English population, from a baseline year of 2015. For closed cohort analyses we started simulations with the population cohort aged 0 in 2015. For open cohort analyses, we started simulations with the 2015 population of children and adolescents (under 18 years) and added new cohorts every five years over the time horizon of analyses. The baseline population numbers were taken from the Office for National Statistics (ONS), by age, sex and quintile of Index of Multiple Deprivation (IMD) [1]. Future cohort numbers (births) were taken from ONS population projections (principal projection) [2]. Since the projections did not account for differences by IMD, we assumed that the proportional distribution of the 0-4 age group population across IMD quintiles in 2015 would remain constant out to 2025.

# Mortality

We derived all-cause mortality rates by age, sex and IMD quintile from ONS counts of population and deaths (S1 Fig) [1]. Numbers were interpolated by single year of age, for modelling, using a smoothing spline model.

S1 Fig All-cause mortality by age, sex and Index of Multiple Deprivation quintile (Q1 is most deprived; Q5 is least deprived)

# Overweight and obesity

Analysis of Health Survey for England data showed BMI to be log-normally distributed. We derived mean and standard deviation of the BMI distribution, by five-year age group, sex and IMD quintile, from the Health Survey for England in 2015. Prevalence of overweight and obesity were determined using BMI cut-offs defined by the International Obesity Taskforce [3]. Base case prevalence is illustrated for children (2-18 years) and adults (≥18 years) in S2 Fig. We excluded children under age two from the BMI analyses due to an absence of BMI data and an absence of evidence on the effects of kilocalorie changes at these ages.


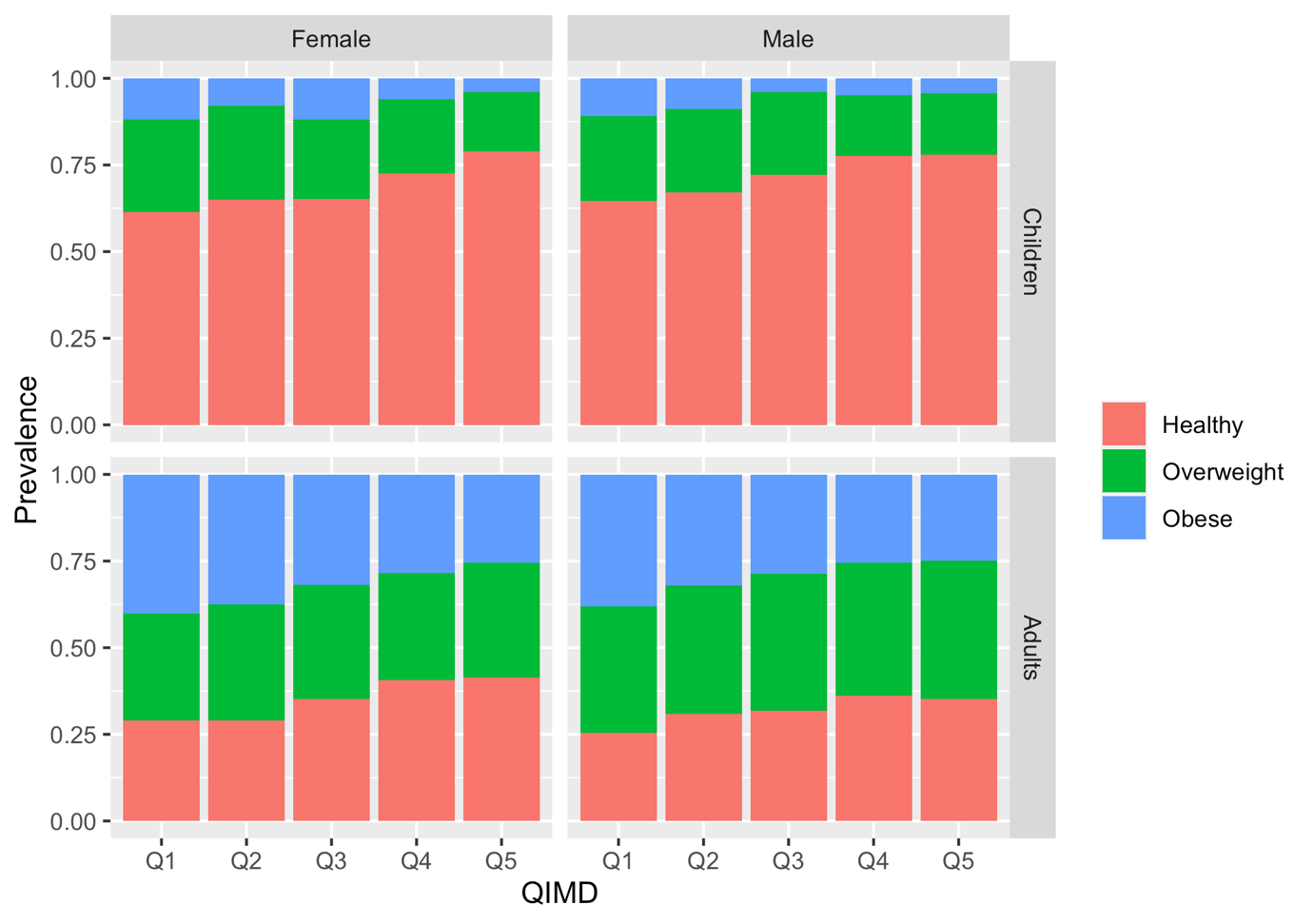


S2 Fig Prevalence of healthy weight, overweight and obesity, in England in 2015, by Index of Multiple Deprivation quintile (Q1 is most deprived; Q5 is least deprived)

## Calculating effect of change in sugar intake

To calculate the effect of change in sugar intake on BMI, we first estimated average body weight in kilograms in 2015 from baseline BMI values and average height, which we derived by age, sex and IMD quintile from the Health Survey for England. Assuming a kilocalorie content of sugar of 3.75 kcal/g [4], we then estimated change in body weight due to the SDIL using energy balance equations derived by Hall et al for children and adults [5,6]:

Boys: $\Delta kg= \frac{\Delta kcal}{68-2.5 \times age}$

Girls: $\Delta kg= \frac{\Delta kcal}{62-2.5 \times age}$

Adults (18+ years): $\Delta kg= \Delta kcal \times\frac{4.184}{100} \times t$

where $t=0.5 \left( year 1 \right), 0.725 \left( year 2 \right), 0.95 \left( year 3 \right), 1 (year 4+)$

Using the new weight and assuming no change in height, we determined the change in mean BMI, by age, sex and IMD quintile. From the change in mean BMI, we then determined the change in shape and scale of the lognormal BMI distribution, due to the SDIL.

The impact of changes in BMI on all-cause mortality was estimated using hazard ratios from a meta-analysis of individual participant data from 239 prospective studies by the Global BMI Mortality Collaboration (S1 Tab) [7].

S1 Tab All-cause mortality hazard ratios^7^

| BMI category | Hazard ratios (95% confidence interval) | |
| --- | --- | --- |
|  | Men | Women |
| 15 to <18.5 | 1.83 (1.66 to 2.02) | 1.53 (1.44 to 1.62) |
| 18.5 to <25 | 1.00 (0.98 to 1.02) | 1.00 (0.98 to 1.02) |
| 25 to <30 | 1.12 (1.11 to 1.13) | 1.08 (1.07 to 1.10) |
| 30 to <35 | 1.70 (1.63 to 1.77) | 1.37 (1.34 to 1.40) |
| 35 to <40 | 2.68 (2.54 to 2.83) | 1.86 (1.79 to 1.93) |
| 40 to <60 | 4.24 (3.77 to 4.76) | 2.73 (2.57 to 2.91) |

# Dental caries

We derived current rates of dental caries in deciduous and permanent teeth, by age and IMD quintile, from data collected in the Children’s Dental Health Survey 2013 [8] and the Adult Dental Health Survey 2009 [9].

In analyses of the Children’s Dental Health Survey, we derived prevalence of any decayed, missing or filled teeth (dmft/DMFT) that were deciduous (dmft>0) or permanent (DMFT>0), and the mean number of dmft or DMFT among prevalent cases.

To derive prevalence and DMFT rates in the Adult Dental Health Survey, we first had to derive DMFT in dentate adults by summing numbers of decayed, missing and filled teeth. Since the adult survey did not record reasons for tooth absence, we adjusted rates to exclude teeth missing for reasons other than dental caries, using estimates from the oral health literature. We carried out this adjustment in two steps.

First, we reduced the mean number of missing teeth to account for congenital tooth absence. Meta-analysis of hypodontia studies suggests that around 7% of Europeans have congenital absence of one or more teeth [10]. From the distribution of number of missing teeth in the European studies, we estimated the mean number of congenitally missing teeth to be 1.95.

Second, we reduced the remaining number of missing teeth to account for extraction due to periodontal disease, trauma, and other causes not related to dental caries. Surveys of dental practices in the UK, suggest that just over half of tooth extractions are due to dental cares, with the exact proportion varying by age (S2 Tab) [11,12].

Finally, having adjusted for unrelated causes of missing teeth in the dentate population, we determined DMFT rates in the whole population by adjusting for the prevalence of edentulism (absence of all teeth), which was recorded in the Adult Dental Health Survey (S3 Fig).

The final rates derived for input to the modelling are shown in S4 Fig (dental caries prevalence) and S5 Fig (dmft/DMFT rates in prevalent cases).

S2 Tab Proportion of tooth extractions due to caries (derived from data in McCaul et al [11])

| Age group (years) | Proportion due to caries |
| --- | --- |
| 0–10 | 45% |
| 11–20 | 38% |
| 21–30 | 85% |
| 31–40 | 65% |
| 41–50 | 55% |
| 51–60 | 39% |
| 61–70 | 45% |
| 71+ | 46% |
| All ages | 55% |


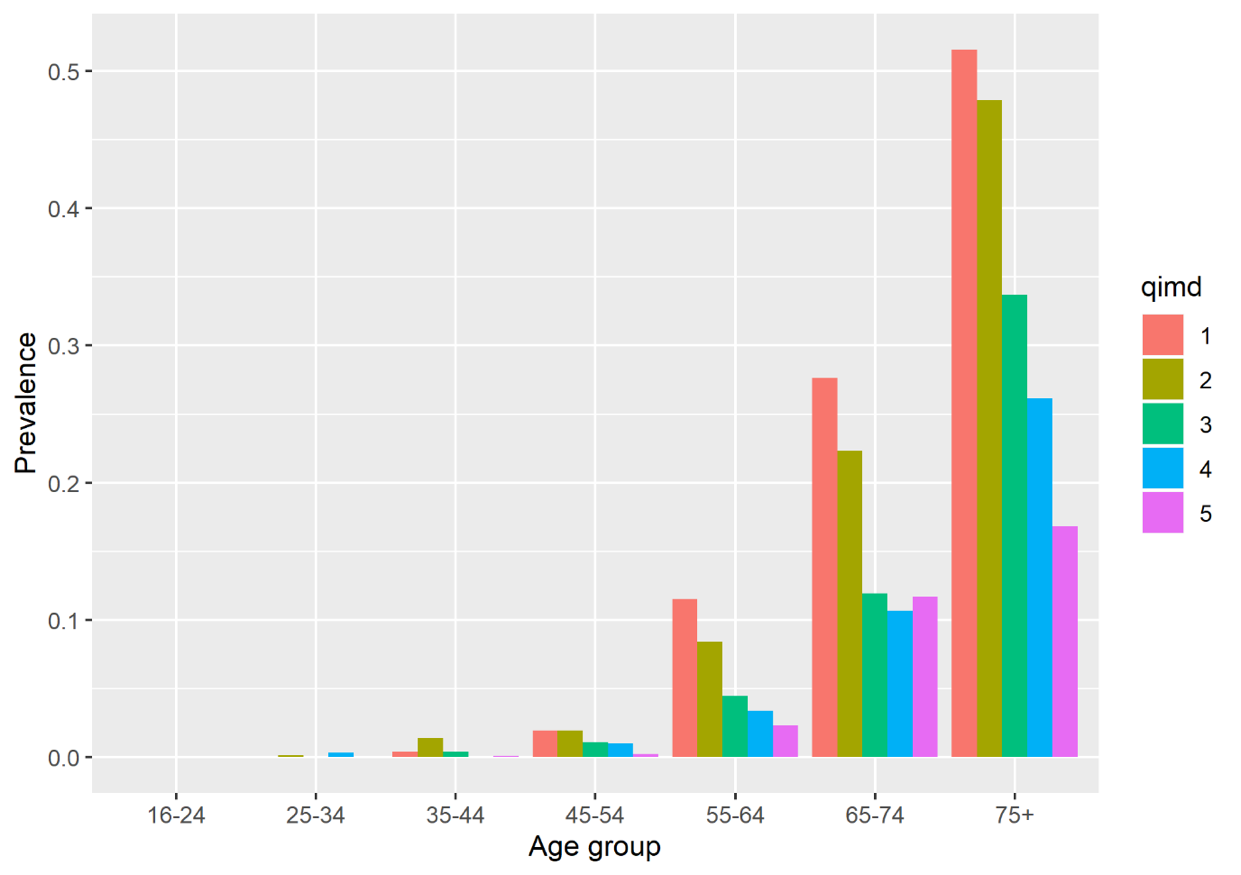


S3 Fig Prevalence of edentulism, by age and Index of Multiple Deprivation quintile (Q1 is most deprived; Q5 is least deprived).


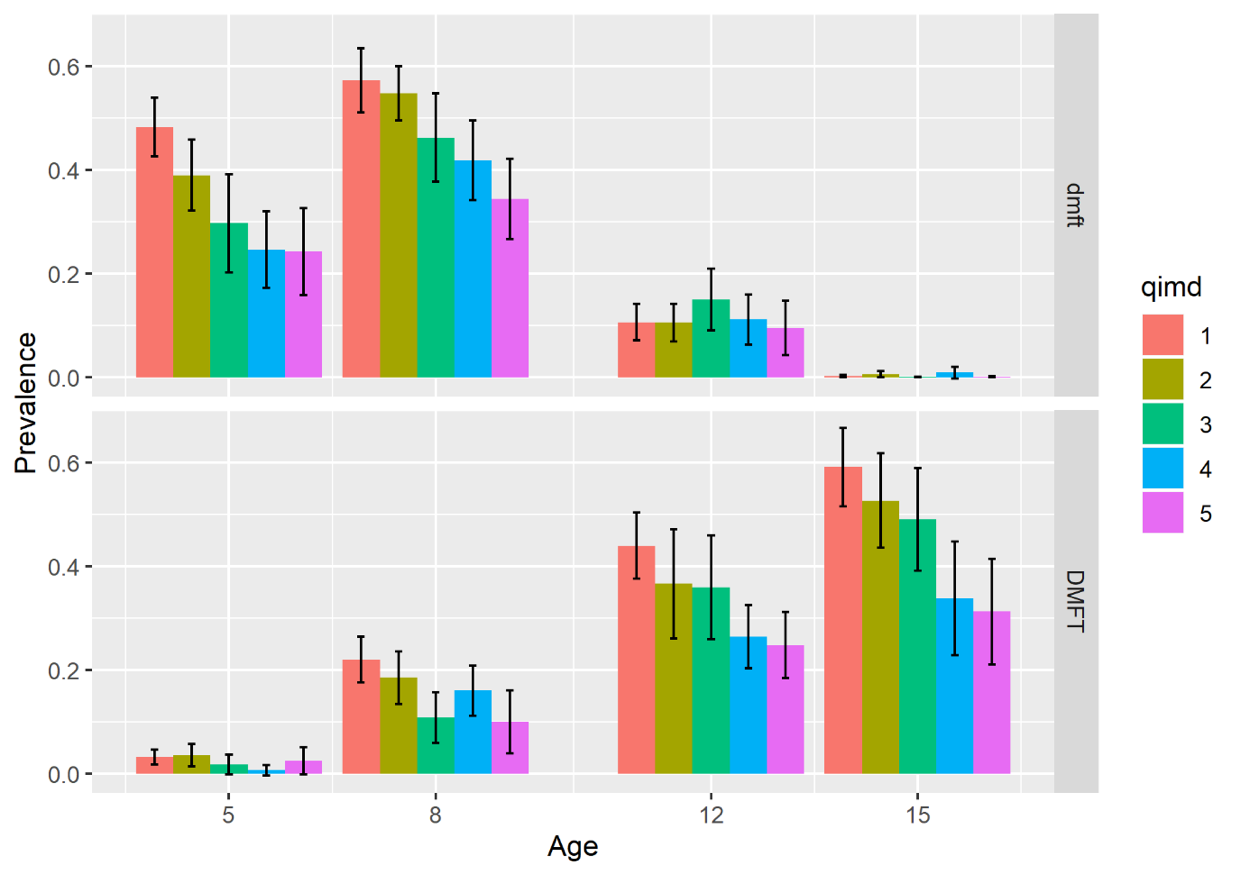


S4 Fig Prevalence of decayed, missing and filled deciduous (dmft>0) and permanent (DMFT>0) teeth, by age at time of survey and Index of Multiple Deprivation quintile (qimd: Q1 is most deprived; Q5 is least deprived. Error bars reflect 95% confidence interval).


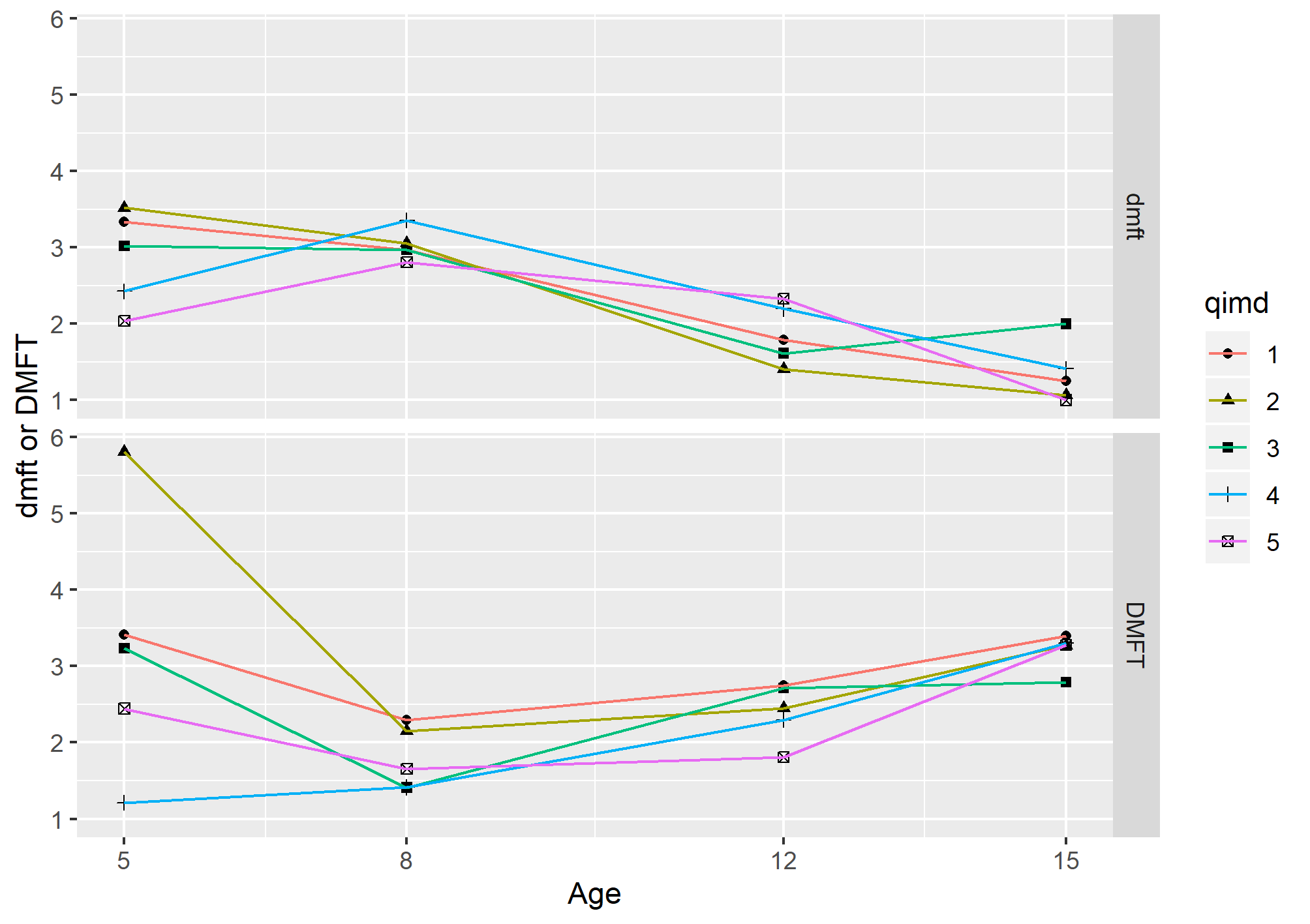


S5 Fig Mean number of decayed, missing and filled deciduous (dmft) and permanent (DMFT) teeth, by age at time of survey and Index of Multiple Deprivation quintile (Q1 is most deprived; Q5 is least deprived).

Calculating effect of change in sugar intake

To calculate the effect of change in sugar intake on dental caries, we applied a dose-response relationship, which we derived from the oral health literature. In a systematic review, Moynihan and Kelly [13] identified 55 studies on the relationship between sugar intake and dental caries. Data from these studies consistently demonstrated a relationship between the amount of sugar consumed and development of dental caries in both children and adults. Variability in metrics prevented meta-analysis and quantification of a dose-response relationship from the studies in the Moynihan and Kelly review. However, a dose-response relationship was estimated in a more recent longitudinal study of sugar consumption and dental caries in Finland [14]. In this analysis of data from three national surveys (2000, 2004 and 2011), Bernabe et al found an average increase of 0.09 DMFT (95% CI: 0.02 to 0.15) for every additional 10 g per day of sugars consumed. For the SDIL analyses, we applied this dose-response relationship to determine change in permanent teeth (DMFT) and assumed the same dose-response relationship for deciduous teeth (dmft), given the consistency of evidence for children and adults in the earlier systematic review.

# Quality of life

We quantified the impact of changes in overweight and obesity and dental caries on quality of life in quality-adjusted life years (QALYs). QALYs were calculated by multiplying lifetable model predictions of years of life lived by quality-of-life weights that range from 0 (death) to 1 (full health). Background quality-of-life was estimated in the model by age, sex and IMD quintile, using a regression of utility weights by Love-Koh et al [15] using the standard European EQ-5D health state measurement instrument in Health Survey for England data.

Calculating effect of change in overweight/obesity and dental caries

Being overweight or obese or having dental caries impacts on quality of life. In model simulations, we adjusted background quality of life based on the changing prevalence of these conditions, due to the SDIL, and the quality of life associated with each condition (S3 Tab).

Utility weights associated with dental caries were taken from a UK catalogue of EQ-5D scores [16]. Utility weights associated with different levels of BMI were sourced separately for children and adults: values for children/adolescents from a meta-analysis of utility studies in populations under 18 years [17]; and values for adults from an EQ-5D study of participants in the Health Survey for England [18].

Since the utility weights were sourced from different studies, we standardised the weights applied in the modelling, using the mean utility weight measured in the associated study population or, in the case of the meta-analysis, using a mean value estimated from the utilities across all BMI categories.

S3 Tab Utility weights applied in the modelling

| Dental caries | Mean utility | 0.828 |
| --- | --- | --- |
|  | Dental caries (ICD9 521.00) | -0.002 (-0.026 to 0.022) |
| BMI ≥18 years | Mean utility | 0.851 |
|  | <18.5 | 0.000 (-0.024 to 0.024) |
|  | 18.5 to <25 | Reference |
|  | 25 to <30 | -0.005 (-0.013 to 0.002) |
|  | 30 to <40 | -0.031 (-0.041 to -0.020) |
|  | 40+ | -0.105 (-0.137 to -0.072) |
| BMI <18 years | Mean utility | 0.844 |
|  | Healthy | 0.007 (0.005 to 0.008) |
|  | Overweight | -0.013 (-0.017 to -0.010) |
|  | Obese | -0.023 (-0.031 to -0.015) |
| NB. Values reflect the mean utility weight and utility increment(s) associated with prevalence of dental caries or category of BMI. | | |

# References

1. Office for National Statistics. Deaths and Populations by sex, age and IMD decile, England & Wales, 2001-2015 2017 [Accessed 8 June 2020]. Available from: <https://www.ons.gov.uk/peoplepopulationandcommunity/birthsdeathsandmarriages/deaths/adhocs/006518deathsandpopulationsbyindexofmultipledeprivationimddecileenglandandwales2001to2015>.

2. Office for National Statistics. 2016-based National Population Projections 2017 [Accessed 1 September 2020]. Available from: <https://www.ons.gov.uk/peoplepopulationandcommunity/populationandmigration/populationprojections/datasets/z3zippedpopulationprojectionsdatafilesengland>.

3. Cole TJ, Lobstein T. Extended international (IOTF) body mass index cut‐offs for thinness, overweight and obesity. Pediatric obesity. 2012;7(4):284-94.

4. FAO. Food energy - methods of analysis and conversion factors. Rome: Food and Agriculture Organization of the United Nations, 2003.

5. Hall KD, Butte NF, Swinburn BA, Chow CC. Dynamics of childhood growth and obesity: development and validation of a quantitative mathematical model. The lancet Diabetes & endocrinology. 2013;1(2):97-105.

6. Hall KD, Sacks G, Chandramohan D, Chow CC, Wang YC, Gortmaker SL, et al. Quantification of the effect of energy imbalance on bodyweight. Lancet. 2011;378(9793):826-37. PubMed PMID: S0140-6736(11)60812-X.

7. Di Angelantonio E, Bhupathiraju SN, Wormser D, Gao P, Kaptoge S, de Gonzalez AB, et al. Body-mass index and all-cause mortality: individual-participant-data meta-analysis of 239 prospective studies in four continents. Lancet. 2016;388(10046):776-86.

8. Office for National Statistics Social Survey Division. Children's Dental Health Survey, 2013. UK Data Service; 2015.

9. Office for National Statistics Social Survey Division. Adult Dental Health Survey, 2009. UK Data Service; 2012.

10. Khalaf K, Miskelly J, Voge E, Macfarlane TV. Prevalence of hypodontia and associated factors: a systematic review and meta-analysis. J Orthod. 2014;41(4):299-316.

11. McCaul L, Jenkins W, Kay E. The reasons for the extraction of various tooth types in Scotland: a 15-year follow up. J Dent. 2001;29(6):401-7.

12. Richards W, Ameen J, Coll A, Higgs G. Reasons for tooth extraction in four general dental practices in South Wales. Br Dent J. 2005;198(5):275-8.

13. Moynihan P, Kelly S. Effect on caries of restricting sugars intake: systematic review to inform WHO guidelines. J Dent Res. 2014;93(1):8-18.

14. Bernabé E, Vehkalahti MM, Sheiham A, Lundqvist A, Suominen AL. The shape of the dose-response relationship between sugars and caries in adults. J Dent Res. 2016;95(2):167-72.

15. Love-Koh J, Asaria M, Cookson R, Griffin S. The social distribution of health: estimating quality-adjusted life expectancy in England. Value Health. 2015;18(5):655-62.

16. Sullivan PW, Slejko JF, Sculpher MJ, Ghushchyan V. Catalogue of EQ-5D scores for the United Kingdom. Med Decis Making. 2011;31(6):800-4.

17. Brown V, Tan E, Hayes A, Petrou S, Moodie ML. Utility values for childhood obesity interventions: a systematic review and meta‐analysis of the evidence for use in economic evaluation. Obes Rev. 2018;19(7):905-16.

18. Maheswaran H, Petrou S, Rees K, Stranges S. Estimating EQ-5D utility values for major health behavioural risk factors in England. J Epidemiol Community Health. 2013;67(2):172-80.
