# Supplementary material for: Impact of the UK soft drinks industry levy on health and health inequalities in children and adolescents in England: An interrupted time series analysis and population health modelling study
Source: PLoS Med. 2024 Mar 28;21(3):e1004371. doi: 10.1371/journal.pmed.1004371 (PMC11008889; doi:10.1371/journal.pmed.1004371)
Supplement: S3 Text — (DOCX) [file pmed.1004371.s003.docx]

# Additional results

S1 Fig Impact of the SDIL on purchased sugar (g/household/week) in soft drinks by IMD quintile (Q1 is most deprived – Q5 is least deprived).


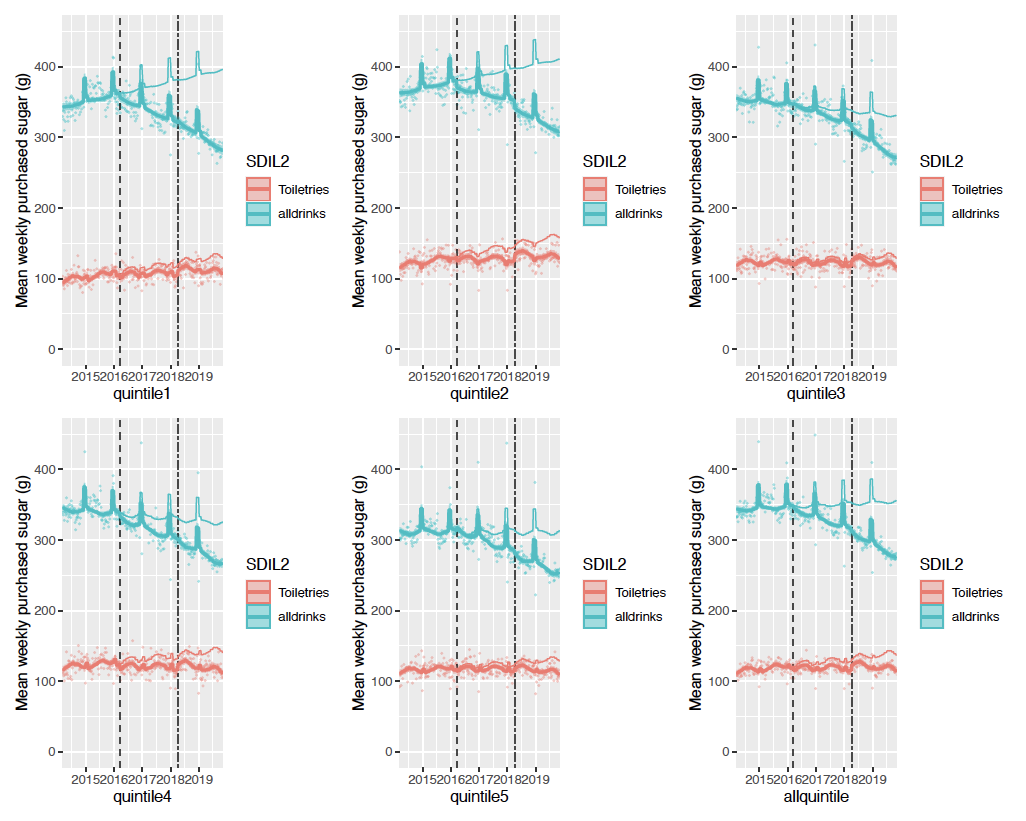


S2 Fig Impact of the SDIL on purchased sugar (g/household/week) in soft drinks by IMD quintile (Q1 is most deprived – Q5 is least deprived) without imputation of missing data.

S1 Tab Absolute change in sugar purchased from drinks when missing data are not imputed.

| IMD | Change of sugar in purchased drinks, compared to counterfactual*, when missing data are not imputed | |
| --- | --- | --- |
|  | Absolute change in g/household/week | Relative change as a percentage |
| Q1 | -114.6 (-107, -121.6) | -28.9 (-30.7, -27.2) |
| Q2 | -102.9 (-96.0, -109.9) | -25.0 (-26.7, -23.3) |
| Q3 | -60.2(-53.7, -66.6) | -18.1 (-20.1, -16.2) |
| Q4 | -58.5(-52.4, -64.7) | -17.9 (-19.8, -16.1) |
| Q5 | -59.1(-53.3, -64.9) | -18.8 (-20.7, -17.0) |
| All quintiles | -79.5(-73.9, -85.0) | -22.3 (-23.9, -20.8) |
| * Counterfactual scenario is based on pre-announcement trends and therefore assumes there has been no announcement or implementation of the SDIL.  NB. Index of Multiple Deprivation (IMD) quintiles: Q1 (most deprived) – Q5 (least deprived). Values are mean and 95% confidence intervals. | | |

S2 Tab Annual average change in dental caries (95% uncertainty interval) between 2015 and 2025, by age group, sex and IMD quintile

| IMD | 2-4 years | 5-7 years | 8-12 years | 13-17 years |
| --- | --- | --- | --- | --- |
| Female | | | | |
| Q1 | -139 (-248 to -36.1) | -245 (-436 to -63.3) | -290 (-517 to -75.1) | -419 (-747 to -108) |
| Q2 | -58.2 (-102 to -15) | -136 (-239 to -34.9) | -174 (-307 to -44.8) | -249 (-438 to -64) |
| Q3 | 9.19 (1.23 to 20.6) | 27.7 (3.7 to 62.2) | 39.4 (5.26 to 88.4) | 60.4 (8.05 to 135) |
| Q4 | -2.49 (-7.65 to 1.31) | -10.6 (-32.7 to 5.58) | -14.5 (-44.7 to 7.62) | -21.5 (-66.1 to 11.3) |
| Q5 | -10.2 (-20.4 to -2.3) | -25 (-50.2 to -5.68) | -35.1 (-70.5 to -7.97) | -63.1 (-127 to -14.3) |
| Male | | | | |
| Q1 | -146 (-261 to -37.8) | -257 (-458 to -66.5) | -305 (-543 to -78.8) | -439 (-782 to -114) |
| Q2 | -61.3 (-108 to -15.7) | -143 (-252 to -36.7) | -183 (-322 to -47) | -261 (-459 to -67.1) |
| Q3 | 9.63 (1.28 to 21.6) | 29 (3.87 to 65.1) | 41.3 (5.51 to 92.6) | 63.1 (8.42 to 142) |
| Q4 | -2.61 (-8.02 to 1.37) | -11.1 (-34.3 to 5.84) | -15.2 (-46.8 to 7.99) | -22.6 (-69.5 to 11.9) |
| Q5 | -10.7 (-21.5 to -2.43) | -26.4 (-52.9 to -5.98) | -37 (-74.3 to -8.39) | -66.5 (-133 to -15.1) |
| NB. Index of Multiple Deprivation (IMD) quintiles: Q1 (most deprived) – Q5 (least deprived) | | | | |

S3 Tab Annual average change e in cases of overweight (95% uncertainty interval) between 2015 and 2025, by age group, sex and IMD quintile

| IMD | 2-4 years | 5-7 years | 8-12 years | 13-17 years |
| --- | --- | --- | --- | --- |
| Female | | | | |
| Q1 | -4,000 (-4,630 to -3,390) | -4,470 (-5,190 to -3,780) | -2,270 (-2,640 to -1,910) | -1,810 (-2,110 to -1,520) |
| Q2 | -2,810 (-3,350 to -2,270) | -3,120 (-3,720 to -2,520) | -1,920 (-2,300 to -1,540) | -1,640 (-1,970 to -1,310) |
| Q3 | 891 (250 to 1,520) | 149 (42.2 to 254) | 393 (111 to 669) | 447 (126 to 761) |
| Q4 | -266 (-661 to 139) | -238 (-596 to 123) | -198 (-496 to 102) | -220 (-550 to 113) |
| Q5 | -780 (-1,150 to -413) | -576 (-847 to -306) | -556 (-820 to -295) | -694 (-1,030 to -366) |
| Male | | | | |
| Q1 | -3,570 (-4,130 to -3,030) | -2,490 (-2,900 to -2,090) | -2,430 (-2,830 to -2,040) | -1,730 (-2,010 to -1,450) |
| Q2 | -2,420 (-2,890 to -1,950) | -2,880 (-3,440 to -2,320) | -1,970 (-2,360 to -1,580) | -1,220 (-1,470 to -982) |
| Q3 | 661 (186 to 1,130) | 685 (193 to 1,170) | 720 (203 to 1,230) | 406 (115 to 691) |
| Q4 | -196 (-486 to 103) | -162 (-407 to 83.7) | -180 (-449 to 92.5) | -197 (-492 to 101) |
| Q5 | -409 (-597 to -219) | -644 (-950 to -341) | -532 (-786 to -281) | -728 (-1,080 to -385) |
| NB. Index of Multiple Deprivation (IMD) quintiles: Q1 (most deprived) – Q5 (least deprived) | | | | |

S4 Tab Annual average change in cases of obesity (95% uncertainty interval) between 2015 and 2025, by age group, sex and IMD quintile

| IMD | 2-4 years | 5-7 years | 8-12 years | 13-17 years |
| --- | --- | --- | --- | --- |
| Female | | | | |
| Q1 | -1,240 (-1,420 to -1,060) | -1,190 (-1,370 to -1,010) | -2,150 (-2,500 to -1,820) | -2,370 (-2,750 to -2,000) |
| Q2 | -936 (-1,110 to -762) | -443 (-526 to -361) | -1,100 (-1,310 to -883) | -1,510 (-1,810 to -1,220) |
| Q3 | 249 (69 to 429) | 309 (87 to 527) | 274 (77.1 to 467) | 312 (87.8 to 532) |
| Q4 | -34 (-84.3 to 18) | -51.1 (-127 to 26.6) | -117 (-293 to 60.5) | -123 (-308 to 63.7) |
| Q5 | -395 (-580 to -211) | -57.7 (-84.4 to -30.8) | -94.4 (-139 to -50.1) | -355 (-524 to -188) |
| Male | | | | |
| Q1 | -881 (-1,010 to -752) | -2,250 (-2,610 to -1,900) | -1,590 (-1,850 to -1,350) | -1,340 (-1,560 to -1,130) |
| Q2 | -1,490 (-1,770 to -1,200) | -712 (-847 to -577) | -757 (-905 to -610) | -906 (-1,080 to -729) |
| Q3 | 207 (57.6 to 355) | 128 (35.9 to 220) | 134 (37.5 to 228) | 160 (45 to 273) |
| Q4 | -9.63 (-23.8 to 5.11) | -104 (-260 to 53.9) | -66.9 (-167 to 34.6) | -48 (-120 to 24.8) |
| Q5 | -18.8 (-27.2 to -10.1) | -211 (-310 to -112) | -187 (-276 to -99.1) | -147 (-216 to -77.7) |
| NB. Index of Multiple Deprivation (IMD) quintiles: Q1 (most deprived) – Q5 (least deprived) | | | | |
