# Supplementary material for: Impact of the UK soft drinks industry levy on health and health inequalities in children and adolescents in England: An interrupted time series analysis and population health modelling study
Source: PLoS Med. 2024 Mar 28;21(3):e1004371. doi: 10.1371/journal.pmed.1004371 (PMC11008889; doi:10.1371/journal.pmed.1004371)
Supplement: Attachment — Submitted filename: Response to reviewers.docx [file pmed.1004371.s004.docx]

-----Original Message-----
From: [em.pmedicine.0.8792e3.fdb938eb@editorialmanager.com](mailto:em.pmedicine.0.8792e3.fdb938eb@editorialmanager.com)<[em.pmedicine.0.8792e3.fdb938eb@editorialmanager.com](mailto:em.pmedicine.0.8792e3.fdb938eb@editorialmanager.com)> On Behalf Of PLOS Medicine
Sent: 23 November 2023 17:13
To: Peter Scarborough <[peter.scarborough@phc.ox.ac.uk](mailto:peter.scarborough@phc.ox.ac.uk)>
Subject: PMEDICINE-D-23-02980R1 Decision - [EMID:f1ce7acb6b7c593f]

Dear Dr. Scarborough,

Thank you very much for submitting your manuscript "Impact of the UK Soft Drinks Industry Levy on health and health inequalities in children and adolescents in England: an interrupted time series analysis and population health modelling study" (PMEDICINE-D-23-02980R1) for consideration at PLOS Medicine. 

Your paper was evaluated by a senior editor and discussed among all the editors here. It was also discussed with an academic editor with relevant expertise, and sent to independent reviewers, including a statistical reviewer. The reviews are appended at the bottom of this email and any accompanying reviewer attachments can be seen via the link below:


In light of these reviews, I am pleased to tell you that we would like to consider a revised version that addresses the reviewers' and editors' comments. Obviously we cannot make any decision about publication until we have seen the revised manuscript and your response, and we plan to seek re-review by one or more of the reviewers. 

In revising the manuscript for further consideration, your revisions should address the specific points made by each reviewer and the editors. Please also check the guidelines for revised papers at [http://journals.plos.org/plosmedicine/s/revising-your-manuscript](https://protect-au.mimecast.com/s/rtnjC1WZxYTpGwYqJF4o8dy?domain=journals.plos.org) for any that apply to your paper. In your rebuttal letter you should indicate your response to the reviewers' and editors' comments, the changes you have made in the manuscript, and include either an excerpt of the revised text or the location (eg: page and line number) where each change can be found. Please submit a clean version of the paper as the main article file; a version with changes marked should be uploaded as a marked up manuscript.

In addition, we request that you upload any figures associated with your paper as individual TIF or EPS files with 300dpi resolution at resubmission; please read our figure guidelines for more information on our requirements: [http://journals.plos.org/plosmedicine/s/figures](https://protect-au.mimecast.com/s/ihIdC2xZzgHk3ow85fxDZR3?domain=journals.plos.org). While revising your submission, please upload your figure files to the PACE digital diagnostic tool, [https://pacev2.apexcovantage.com/](https://protect-au.mimecast.com/s/J3AmC3Q8BjImKYQ2DU5JJgi?domain=pacev2.apexcovantage.com). PACE helps ensure that figures meet PLOS requirements. To use PACE, you must first register as a user. Then, login and navigate to the UPLOAD tab, where you will find detailed instructions on how to use the tool. If you encounter any issues or have any questions when using PACE, please email us at [PLOSMedicine@plos.org](mailto:PLOSMedicine@plos.org).

We expect to receive your revised manuscript by Dec 14 2023 11:59PM. Please email us ([plosmedicine@plos.org](mailto:plosmedicine@plos.org)) if you have any questions or concerns.

***Please note while forming your response, if your article is accepted, you may have the opportunity to make the peer review history publicly available. The record will include editor decision letters (with reviews) and your responses to reviewer comments. If eligible, we will contact you to opt in or out.***

We ask every co-author listed on the manuscript to fill in a contributing author statement, making sure to declare all competing interests. If any of the co-authors have not filled in the statement, we will remind them to do so when the paper is revised. If all statements are not completed in a timely fashion this could hold up the re-review process. If new competing interests are declared later in the revision process, this may also hold up the submission. Should there be a problem getting one of your co-authors to fill in a statement we will be in contact. YOU MUST NOT ADD OR REMOVE AUTHORS UNLESS YOU HAVE ALERTED THE EDITOR HANDLING THE MANUSCRIPT TO THE CHANGE AND THEY SPECIFICALLY HAVE AGREED TO IT. You can see our competing interests policy here: [http://journals.plos.org/plosmedicine/s/competing-interests](https://protect-au.mimecast.com/s/p_oJC4QZEkIJ5A1l4cqXR9S?domain=journals.plos.org).

Please use the following link to submit the revised manuscript: 

[https://www.editorialmanager.com/pmedicine/](https://protect-au.mimecast.com/s/fXn_C5QZJlI09YG6kuPP1H6?domain=editorialmanager.com)

Your article can be found in the "Submissions Needing Revision" folder. 

To enhance the reproducibility of your results, we recommend that you deposit your laboratory protocols in protocols.io, where a protocol can be assigned its own identifier (DOI) such that it can be cited independently in the future. Additionally, PLOS ONE offers an option to publish peer-reviewed clinical study protocols. Read more information on sharing protocols at [https://plos.org/protocols?utm_medium=editorial-email&utm_source=authorletters&utm_campaign=protocols](https://protect-au.mimecast.com/s/S-xuC6X1LmSomlKyLUwENF7?domain=plos.org)

Please ensure that the paper adheres to the PLOS Data Availability Policy (see [http://journals.plos.org/plosmedicine/s/data-availability](https://protect-au.mimecast.com/s/hKZJC71ZNnhmNjkZGUGcFV6?domain=journals.plos.org)), which requires that all data underlying the study's findings be provided in a repository or as Supporting Information. For data residing with a third party, authors are required to provide instructions with contact information for obtaining the data. PLOS journals do not allow statements supported by "data not shown" or "unpublished results." For such statements, authors must provide supporting data or cite public sources that include it. 

We look forward to receiving your revised manuscript. 

Best wishes,
Pippa

Philippa Dodd, MBBS MRCP PhD
-----------------------------------------------------------
COMMENTS FROM THE ACADEMIC EDITOR
Some of the queries point out fairly serious error, e.g using areal data to ascribe to each household Socio-economic status. I think this calls for a major revision. Some of the comments are not minor. If they address all the comments, we can then make a decision.

COMMENTS FROM THE EDITORS
GENERAL
Please respond to all editor and reviewer comments detailed below in full.
We understand the need to update the ITSA and to stratify by additional parameters to answer the specific question posed here. We agree with the statistical reviewer (please see below) that from a novelty perspective, it would be helpful to understand the rationale for the ITSA analysis destined for the BMJOpen as well as that presented here. Was the former to set a precedent for the (slightly updated) methodology used here, for example? Please provide details and please incorporate your response into the manuscript as necessary.

DATA AVAILABILITY STATEMENT
Thank you for including a statement pertaining to your data availability. The Data Availability Statement (DAS) requires revision. For each data source used in your study: 
a) If the data are freely or publicly available, note this and state the location of the data: within the paper, in Supporting Information files, or in a public repository (include the DOI or accession number).
b) If the data are owned by a third party but freely available upon request, please note this and state the owner of the data set and contact information for data requests (web or email address). Note that a study author cannot be the contact person for the data.
c) If the data are not freely available, please describe briefly the ethical, legal, or contractual restriction that prevents you from sharing it. Please also include an appropriate contact (web or email address) for inquiries (again, this cannot be a study author).
We think that point c) would be most applicable here.

- The updated Data Availability Statement is as follows: “The data that support the PRIMEtime model are freely available from the following sources:
  - Global Burden of Disease Project (https://vizhub.healthdata.org/gbd-results/)
  - Office for National Statistics (https://vizhub.healthdata.org/gbd-results/)
  - Health Survey for England (https://digital.nhs.uk/data-and-information/publications/statistical/health-survey-for-england)
  - Children’s Dental Health Survey 2013 (https://digital.nhs.uk/data-and-information/publications/statistical/children-s-dental-health-survey/child-dental-health-survey-2013-england-wales-and-northern-ireland)
  - Adult’s Dental Health Survey 2009 (https://digital.nhs.uk/data-and-information/publications/statistical/adult-dental-health-survey/adult-dental-health-survey-2009-summary-report-and-thematic-series)
- The interrupted time series analyses uses commercial data purchased from the Kantar Fast Moving Consumer Goods Panel, and cannot be shared without permission from the data owners (Kantar).”

COMPETING INTERESTS
All authors must declare their relevant competing interests per the PLOS policy, which can be seen here:
[https://journals.plos.org/plosmedicine/s/competing-interests](https://protect-au.mimecast.com/s/rQWuC81ZPohjq8RYNCY0vXg?domain=journals.plos.org)
For authors with ties to industry, please indicate whether any of the interests has a financial stake in the results of the current study.
Please add this statement to the manuscript's Competing Interests: "JA is an Academic Editor on PLOS Medicine's editorial board."

- The competing interests statement has been amended as suggested.

ETHICS STATEMENT
As your study utilizes human participant data, we think an ethics statement would be appropriate.

- Our study only uses secondary analyses of data from various data sources, including the Global Burden of Disease project which is based on hundreds of datasets worldwide. It is impractical to provide a reference to all of the ethics statements that cover the priomary data collection for the studies whose datasets we have used in this project. Therefore, we disagree and have not provided an ethics statement.

ABSTRACT
Thank you for formatting your abstract according to PLOS Medicine’s style. 
Abstract methods and findings:
Please include the study design, population and setting, number of participants, years during which the study took place, length of follow up, and please explicitly state the main outcome measures.
Please provide (brief) details of the databases used to leverage your data.
Please quantify the main results with 95% CIs (or UIs in this case) and p values. When reporting p values please report as p<0.001 and where higher the exact p value as p=0.002, for example. If not reporting p values, for the purpose of transparent data reporting please clearly state the reasons why not.
Please include any important dependent variables that are adjusted for in the analyses.
In the last sentence of the Abstract Methods and Findings section, please describe the main limitation(s) of the study's methodology.

Abstract Conclusions:
Please address the study implications without overreaching what can be concluded from the data; the phrase "In this study, we observed ..." may be useful.
Please interpret the study based on the results presented in the abstract, emphasizing what is new without overstating your conclusions.
Please avoid vague statements such as "these results have major implications for policy/clinical care". Mention only specific implications substantiated by the results.
Please avoid assertions of primacy ("We report for the first time....") Line 49 – suggest ‘could’ instead of ‘will’.

- ‘will’ has been replace by ‘could’ in the final sentence.

AUTHOR SUMMARY
At this stage, we ask that you include a short, non-technical Author Summary of your research to make findings accessible to a wide audience that includes both scientists and non-scientists. The authors summary should consist of 2-3 succinct bullet points under each of the following headings:
• Why Was This Study Done? Authors should reflect on what was known about the topic before the research was published and why the research was needed.
• What Did the Researchers Do and Find? Authors should briefly describe the study design that was used and the study’s major findings. Do include the headline numbers from the study, such as the sample size and key findings. 
• What Do These Findings Mean? Authors should reflect on the new knowledge generated by the research and the implications for practice, research, policy, or public health. Authors should also consider how the interpretation of the study’s findings may be affected by the study limitations. In the final bullet point of ‘What Do These Findings Mean?’, please describe the main limitations of the study in non-technical language.
The Author Summary should immediately follow the Abstract in your revised manuscript. This text is subject to editorial change and should be distinct from the scientific abstract. Please see our author guidelines for more information: [https://journals.plos.org/plosmedicine/s/revising-your-manuscript#loc-author-summary](https://protect-au.mimecast.com/s/T-QjC91ZRphmqVB24URni6o?domain=journals.plos.org)

- An author summary has been added following the Abstract.

METHODS and RESULTS
Of all authors who submit modelling studies we ask that the following points are included in the main manuscript. 
Please review the list below, derived from Geoffrey P Garnett, Simon Cousens, Timothy B Hallett, Richard Steketee, Neff Walker. Mathematical models in the evaluation of health programmes. (2011) Lancet DOI:10.1016/S0140-6736(10)61505-X and ensure that each item is included:

* Please provide a diagram that shows the model structure, including how the disease natural history is represented, the process and determinants of disease acquisition, and how the putative intervention could affect the system.

- We have a flow diagram of the modelling calculations in the main manuscript (Figure 1), and we have added a diagram of the state-transition model to the supplementary file Text S2.

*Please provide a complete list of model parameters, including clear and precise descriptions of [the meaning of each parameter, together with the values or ranges for each, with justification or the primary source cited, and important caveats about the use of these values noted].

- We have added a supplement file (Text S1) with details of the model specification for the interrupted time series analysis.
- The lifetable model methods are described and each input parameter is referenced in the main manuscript (Line 191-269). In the supplementary file Text S2 we provide further detail on the lifetable model, and include a sub-section for each input parameter, where we describe the source of the parameter data, including any assumptions made in incorporating these values into the lifetable model, and provide graphs/tables of the input data.

*Please provide a clear statement about how the model was fitted to the data [including goodness-of-fit measure, the numerical algorithm used, which parameter varied, constraints imposed on parameter values, and starting conditions].

- This is not relevant to the lifetable model.

*For uncertainty analyses, please state the sources of uncertainties quantified and not quantified [can include parameter, data, and model structure].

- The parameters included in the uncertainty analyses are described at line 262-263, along with a reference to the supplement TextS1, which presents the data for the relevant parameters. We additionally discuss which sources of unceratinty are included or excluded (e.g. model structure uncertainty) in a paragraph on study limitations in the Discussion (line 451-457)

*Please provide sensitivity analyses to identify which parameter values are most important in the model. Uncertainty estimates seek to derive a range of credible results on the basis of an exploration of the range of reasonable parameter values. The choice of method should be presented and justified.

- We have included sensitivity analysis, starting at Line 263

*Please discuss the scientific rationale for this choice of model structure and identify points where this choice could influence conclusions drawn. Please also describe the strength of the scientific basis underlying the key model assumptions.

- The implications of model choices, including assumptions embedded in the interrupted time series analysis approach, the household purchasing data that were used in the interrupted time series analysis, and the lifetable model structure and parameters, are discused in the paper at lines 417-458
- In addition, the supplement Text S2 provides an explanation of the lifetable modelling approach and the data and decisions that went into designing each of the model components.

Line 117 – should ‘MD’ be ‘IMD’ at the end of this line?

- We have added the missing 'I’.

Please also see reviewer comments (below) regarding additional details required in respect of your methodology, which we agree with.

TABLES
Throughout please ensure that all tables are affiliated to an appropriate title and caption which clearly describes their content without the need to refer to the text.

- Checked

Throughout, please ensure that all abbreviations including those used for statistical reporting are clearly defined for the reader.

- Checked

Throughout, please clearly define the numerical values within the tables including those within brackets.

- Checked. Added explanation of brackets in Table 4 note.

Throughout please indicate whether your analyses are adjusted (or not) and in the event that adjusted analyses are presented please detail the factors adjusted for (in an appropriate footnote/caption) and to help facilitate transparent data reporting, please also present the unadjusted analyses for comparison.

- NA

Throughout tables could be presented a little more accessibly and informatively for the reader. For example, when referring to means there are no units of measurement but some refer to weight, others to total number. This could be included in the columns headers for example, ‘mean sugar (g)’ presumably?

- We have added ‘number’ to column headings in Table 2 and ‘Number of quality-adjusted life years’ to Table 3. Tables 1 and 4 already have units of measurement.

Suggest separating upper and lower CI bounds with commas to conserve space and prevent data being split across rows.

- ‘ to ‘ has been replace with ‘, ‘ in all tables

PLOS Medicine requires that where 95% CIs are reported, p values are also reported, please include and report as >0/001 and where higher the exact p value as 0.002, for example. If not reporting p values, for the purpose of transparent data reporting, please clearly state the reasons why not.

- We report 95% uncertainty intervals from Monte Carlo analyses, so p values are not relevant.

FIGURES
Throughout please cite figures (and tables) as outlined here [https://journals.plos.org/plosmedicine/s/figures#loc-how-to-submit-figures-and-captions](https://protect-au.mimecast.com/s/NYDLC0YZv2S2B03mNuPiq0F?domain=journals.plos.org)
Throughout (including the supporting information) please consider avoiding the sue of green and/or red so as to make your figures more accessible to those with color blindness.

- Checked

Please ensure that all figures are affiliated to an appropriate title and caption that clearly describes the figure content without the need to refer to the text. Please ensure that any abbreviations including those used for statistical reporting are clearly defined for the reader.

- Checked

Figure 2 – please include a caption. Please clearly state the meaning of the different colour shading. Please clearly define the meaning of the dots and the lines for the reader.

- We have expanded the Figure 2 captions as follows: “Change in slope index of inequality for life expectancy(purple) and quality-adjusted life expectancy (blue). The shading reflects the distribution of the results, the dot symbol shows the mean, and the error bars show the 95% uncertainty interval.”

DISCUSSION
Please remove all sub-headings form the discussion such that it reads as continuous prose and please ensure that you organize the Discussion as follows: a short, clear summary of the article's findings; what the study adds to existing research and where and why the results may differ from previous research; strengths and limitations of the study; implications and next steps for research, clinical practice, and/or public policy; one-paragraph conclusion.

- We have removed the sub-headings and checked the paragraphs follow the recommended order.

Please remove the financial disclosure and competing interests statement from the end of the discussion and include only in the manuscript submission form when you re-submit your manuscript. In the event of publication, this information will be compiled as metadata.

- Removed

REFERENCES
For in text reference callouts please remove the spaces between different citations, for example line 67 should read ‘[3,4]’.
In the bibliography please ensure that you list up to but no more than 6 author names followed by et al.
For all web references please ensure you include an, ‘Accessed [date].’
Journal name abbreviations should be those listed in the National Center for Biotechnology Information (NCBI) databases.

- References have been updated as indicated

SUPPORTING INFORMATION
Please ensure that you provide titles and legends for each individual table and figure in the Supporting Information which follow the guidance detailed above for the main manuscript.

- Checked

In the published article, supporting information files are accessed only through a hyperlink attached to the captions. For this reason, you must list captions at the end of your manuscript file. You may include a caption within the supporting information file itself, as long as that caption is also provided in the manuscript file. Do not submit a separate caption file.

- Supporting information file captions have been listed at the end of the manuscript

Please cite your Supporting Information as outlined here: [https://journals.plos.org/plosmedicine/s/supporting-information](https://protect-au.mimecast.com/s/HJtKCgZo1xHly52GEUGb8P3?domain=journals.plos.org)
For in-text reference callouts please place citations in square parentheses separate by commas. For example, [1,3,6] or [1-3]. Please check and amend throughout all sub-sections of the manuscript and supporting files.

- References have been updated

Please ensure that other referencing follows the format as detailed above for the main manuscript.

- Checked

As for the main manuscript where reporting 95% CIs please also report p values as detailed above, if not please clearly state the reasons why not.

- NA

COMMENTS FROM THE REVIEWERS:

**Reviewer #1**: The paper models the potential on BMI and dental caries that occurred due to small reductions in sugar from beverages in UK households following a levy. The initial findings are promising that the intervention will effectively achieve increases reductions in both BMI and dental carries. While there are limitations to the methodology in that real dietary intake was not measured, the authors have described these in the limitations section. The analyses does not measure the impact on purchases not brought into the home, as sugary drinks are often consumed outside of the home, the authors may with to comment on this, particularly in relation to teenagers given the high intake of sugar in this age group.

- We have expanded the discussion of out-of-home drink purchases as follows (line 443-450): “Our purchasing data included all drink purchases that were brought into the home, but does not include drinks that were purchased and consumed outside of the home. It is estimated that 10-12% of expenditure on cold non-alcohol beverages in the UK is spent on out-of-home purchases [66], and it is possible this may affect some age groups more than others (e.g. teenager may be more likely to purchase drinks outside of the home). If the SDIL provoked a similar reduction in purchasing of these out-of-home drinks, then we will have under-estimated the full effect of the levy – however, more work is needed to establish this.”

**Reviewer #2**: Overview
This paper uses interrupted time series analyses and lifetable modelling to evaluate the effects of the UK soft drink industry levy on changes in sugar consumption, BMI, dental caries and QALYs in children and adolescents. This is an important topic, and the authors provide strong justification for their study. The paper is interesting, well written and thorough. I have made some minor comments and suggestions.

Comments

Introduction: Says that drinking with <0.5% ABV are exempt, I think this should be >1.2% ABV.

- We have clarified the exemption, starting at Line 85: “…low-alcohol drinks (zero alcohol versions of drinks that are marketed as specific alternatives to high alcohol drinks, or drinks that have had alcohol removed from them until they have got less than 1.2% alcohol by volume)…”

Results: I might have missed them, but I cannot see the full time series model specification and results in the main document or supplementary materials. It would be helpful to include these.

- We have added a supplementary document with the model specficiation (Text S1). In addition to the results in Table 1, we have included a plot of the results for each IMD model in the supplement Text S2 (Figure S1).

Discussion: This is excellent, and clearly explains every key limitation of the study that I noted from the methods.

Typo: MD line 117.

- This has been corrected to ‘IMD’

Note for editor: Thank you for the opportunity to review this paper. I would just like to note that I was unable to review the lifetable model in detail as I am not familiar with these methods. To the best of my knowledge, the methods appear robust.


**Reviewer #3**: Thank you to the authors for submitting this ambitious paper on a clearly very important topic - I enjoyed reading it. Although very well written, my overall opinion is that the paper is trying to do too much. As a consequence of this, several parts of the paper require far more explanation and detail. I also have concerns about some of the methods used and the similarity to another paper by most of the same authors. I feel as though the paper does have the potential for publication in this journal, however in my opinion a number of relatively substantive edits are needed. 

My comments are organised in the order in which they appear in the manuscript, and I have noted whether they are 'major' or 'minor'.

Line 62: Reference to the Rogers et al unpublished paper. Do the authors expect this paper to be published imminently? (Minor)

- The paper has now been published and we have updated the reference

Lines 81 - 83: This sentence explaining the results from the 2016 systematic review is a bit confusing. It implies that the studies included in the review had one of two (quite distinct) outcomes. I would like another sentence or two explaining the mechanisms behind these findings from the authors (Minor)

- We have amended the paragraph as follows: “A 2016 systematic review of the effect of SSB taxes on socioeconomic differences in health concluded that a tax on SSBs would likely lead to changes in population weight of a similar magnitude for all socio-economic groups or slightly better outcomes for lower compared to higher socio-economic groups [22]. Additionally, they concluded that SSB taxes would likely be financially regressive to a small degree (up to 1% or 0.6% of annual household income for low- and high-income households respectively).”

Lines 100 - 103: From reading this, it seems as though the only difference between ITS analysis in the Rogers et al unpublished study and this present study is the fact that you stratified by IMD and used Great Britain rather than England. These analyses are very similar, and to me could have quite easily been sensitivity analysis in the Roger et al unpublished study. I'm not sure these two very similar analyses can be justified as two separate papers (Major)

- This paper focuses on modelling the inequality implications of the SDIL. To get area-level specific results for the model, we had to stratify the interrupted time series analyses by IMD quintile. We can only do this for England, not the whole of the UK (which is what the other paper covers), so it was necessary to redo the interrupted time series analyses for this paper.

Lines 114 - 115: I'm not 100% convinced by the choice of control - has this been used in any other studies aside from the Rogers et al unpublished study? Are there any other alternative choices of control that could have been used? (Minor)

- We have added the following sentence (line 149) to clarify our use of toileteries as a control: “We included weekly household purchasing of toiletries (liquid soap, shampoo, and hair conditioner) as a non-equivalent control group to account for background trends in household purchases. Toiletries were selected as a suitable control group because purchasing is unlikely to be influenced by seasonality or counfounders, including socioeconomic position”

Lines 117 - 188: The Kantar dataset needs to be explained in a lot more detail (Major)

- We have provided additional information in the paragraph describing the Kantar data, starting line 139. It now reads: “The analyses used household purchasing data collected between March 2014 and November 2019 by Kantar Fast Moving Consumer Goods panel, a market research company. In this dataset, participating households are asked to scan the barcodes of food and drink purchases that are brought into the home. These purchasing data are uploaded weekly and linked to data on nutritional content. Household demographic information is updated annually, and participating households receive gift vouchers equivalent to £100 ($122; €112) annually. The panel data are in the form of a line list of purchases rather than a household study. Proprietary weights (called grossupweight) are applied at the level of the purchases rather than the households. Other information attached to the line list of purchases includes the week of purchase, product descriptions, the amount of sugar in grams per 100mls, overall volume and a unique household identifier.”

Lines 117 - 188: The levels of missing data need to be stated in order to get an idea of the extent of the issue (Major)

- The level of missing data changed across the study and was particularly high in the pre-announcement period but this changed substantially over time. Please see the response to Reviewer 4 below for further discussion of missing data.

Line 130: I'm a bit confused by the weights. Could you please elaborate on exactly how they were used?

- The weights used for the analysis were proprietary grossing up weights, created by Kantar Worldpanel. They were used throughout all of our analysis to extrapolate from the size of the panel to the size of the population in Great Britain (GB). For example, weekly household purchases were calculated as sum of all (sugar concentration * volume * Kantar weight)/number of households.

Line 139: Maybe it's my memory failing me, but I don't recall any widespread changes to consumer behaviour in December 2019 due to Britain's exit from the EU. It's not a big issue because COVID came soon after and this is clear justification for your cut-off point, but it seems a bit odd to me (Minor)

- There was some evidence of changes to purchasing behaviour just prior to Brexit (largely brought on my unhelpful media reporting) [1].

Line 147: I understand your use of the AIC to choose the optimal model, but I would like a bit more explanation regarding the number and type of different models you used when selecting your model.

- As mentioned in response to an earlier comment, this study involved redoing interrupted time series analyses previously published for the whole of Great Britain [2], but limiting the analyses to England where we had specification of IMD quintiles.
- In our manuscript, we have provided reference to the previous interrupted time series analyses for a detailed rationale for the model that is used. Our aim for this study, was to replicate the interrupted time series analysis methods as closely as possible, so that the results would be directly comparable.

Lines 157 - 166: I'm really not convinced by the life table modelling approach. Obesity (especially child/adolescent obesity) is a particularly difficult clinical area to model, with lifetime state transition models commonly used. I think there needs to be far more justification about why this highly simplified model structure is appropraite for this complex issue (Major)

- Rather than modelling the population cohorts transitioning through states of overweight, obesity, etc. we model a shift in the mean of the BMI distribution for each age/sex cohort. The change in mean BMI is determined from the reduction in calories from sugar derived in the ITS analyses of the SDIL. As the distribution shifts, we get a changing proportion of children/adolescents below the overweight, obesity, etc. BMI cut-offs (this is further described in the ‘Overweight and Obesity’ section in Text S2).
- The states of the lifetable model only capture the transition between alive and dead (see added model diagram – Figure S1 in Text S2). We quantify impact of the changing prevalence of overweight, obesiity, etc. on the mortality risk in a population impact fraction calculation, using hazard ratios of the association between five BMI categories and mortality (Table S1 in Text S2). These hazard ratios, from the Global BMI Mortality Collaboration, only quantify the hazard from age 20 years, so we do not assume any BMI impact on mortality in the children and adolescent years (although we do calculate quality of life impacts at these ages). There a a number of other published studies that have also modelled public health interventions (including obesity interventions) using life table models in this way [3-5].

Figure 1: I'm confused about how exactly you measured the impact of both BMI and Dental Caries on Quality of Life. Did you about for the fact that there may be an interactive effect? (Minor)

- We calculated the combined QALY impact of BMI and dental caries in a multiplicative way, i.e. rather than add the utilities together (which could theoretically lead to a combined utility greater than 1) we took the product of the utility values.
- It is theoretically possible that the utilities associated with having dental caries and high BMI are not entirely independent, however, the values we have, while derived using the same measures, are by necessity from different studies. We are not aware of any data where such an interaction effect has been explored or quantified.

Lines 202 - 203: I appreciate the use of the EQ-5D to measure utility, however I think the authors need to state up front that these utility weights are based on a sample of adults rather than children. Although the EQ-5D-Y has been developed, there are no current population norms for either the 3L or 5L version, let alone utility scores by age, sex and IMD quintile (Major)

- To be clear, we haven’t used the EQ-5D in our study, but we do rely on studies that have used the EQ-5D to derive the utility values that we then use to adjust the years of life lived for quality of life.
- We have added the following text to the Discussion (starting Line 462): “Finally, in the quality-adjustment of life expectancy and years of life lived, we rely on published estimates of utility values to reflect quality of life associated with having dental caries or obesity, and to reflect the average background quality of life at different ages, sex and IMD quintiles. The development and use of measurement instruments, such as the EQ-5D is well-established in the adult population, but there is far less evidence relating to the experience of children and adolescents. In their systematic review of obesity-related utility values in children and adolescents, for example, Brown et al [48] highlighted the dearth of relevant studies and the resulting high heterogeneity in meta-analysis results. While the uncertainty in child and adolescent utility values does not affect our modelled estimates of dental caries numbers, obesity prevalence or life expectancy, our estimates of *quality-adjusted* life years and *quality-adjusted* life expectancy should be interpreted with more caution.”

"

Line 203: I think it could be worth noting that the health state utilities gathered from reference number 49 and 50 are pretty out of date (Minor)

- The two references (from 2011 and 2013) are somewhat dated, but they are the most recent data available for these condition in the population of England/UK.

Lines 214-215: Need far more explanation about how exactly the life table model was implemented (Major)

- We have added further information on the model to the main manuscript, in accordance with journal requirements (see above). There is also additional detail on the model and input data in the supplementary file (Text S2).

Line 219-220: Can the authors comment on the plausibility of the changes in sugar purchased being maintained over the life course? Is there any evidence to back up this assumption? (Minor)

- The SDIL and other ‘soda taxes’ are a relatively recent intervention, so long-term follow-up is limited. Follow-up of SSB taxes in Mexico and Spain suggest that the effects on consumption were greater at 2 to 3.5 years after implementation; and studies of tobacco taxes (which have been around much longer) suggest that the long-run responses to change in price are greater than in the short-term.
- However, the SDIL, is slightly different in that it is a tiered levy targeted at manufacturers, and has led to reformulation of drink products. It is possible that a different government might repeal the legislation, which could see manufacturers reformulate back to unhealthier products. If this was to occur, then the impact of the SDIL on the inequality in life expectancy in England would be smaller.
- We have highlighted the assumption around a sustained sugar reduction effect and the lack of evidence on which to base this decision as a weakness of our study in the Discussion (Line 455-458)

Line 226 - 228: This linear regression model needs far more explanation. What was the exact model specification? What was the exact sample it was run on? (Major)

- The model was specified as LE ~ IMD, where the outcome variable (LE) was life expectancy (or quality-adjusted life expectancy) in years, and the predictor (IMD) was the Index of Multiple Deprivation divided into quintiles. The regression coefficient reflected the Slope Index of Inequality. We have added these details to this sentence at Line 257-258.
- The life expectancty and quality-adjusted life expectancy data for each IMD quintile were outputs from the lifetable model. Since the lifetable model was run 5000 times for the probabilistic sensitivity analyses, we ran the linear model fitting 5000 times. We then determined the mean and 95% uncertainty interval of the regression coefficients.
- These are the results shown in Figure 2. The data are the slope index of inequality for life expectancy (purple) and quality-adjusted life expectancy (blue). The shading reflects the distribution of the data, the dot symbol shows the mean, and the error bars show the 95% uncertainty interval. We have now included this information in the caption for Figure 2.

Figure 2: I this figure difficult to interpret - it needs more explanation.

- See previous response

Line 309: The headline figure of "19,500 QALYs" is certainly eye catching, but I think it needs to be put into context (in relation to the child/adolescent population in England/UK). I'm not sure whether this is a small or large relative effect! (Major)

- It equates to an average of 0.56 additional quality-adjusted life years per 1000 children and adolescents. We have this clarification at Line 340)

Lines 320-321: As before, I think more justification is needed for two separate papers (Minor)

- See previous response

Line 377: I think you could also discuss the representativeness of the dataset to the English/UK population - how big is the internal vs external validity trade off in this case? (Minor)

- We used the proprietary weighs from Kantar throughout the interrupted time series analyses to demographically weight the panel to reflect Great Britain, and a grossing up factor which increases the size of the panel to the size of the population of Great Britain. This is a consistent weight applied to all purchasing in a given monthly period.

**Reviewer #4**: The study analyzes changes sugar purchased associated with the UK soft drink industry levy and modeled the effect on health in children and adolescents. The study is very relevant but needs to be revised.

I first recommend to change "effects or impact" by changes in or associations as in the absence of an experimental design, causal effects cannot be claimed for both the empirical estimation on sugar purchased and the model to estimate changes in dental caries and body mass index.

- We have changed ‘effect’ to ‘simulated effect’ where it relates to the outputs of the lifetable model.

I understand that toiletries can be an adequate control but the authors should provide a convincing justification. It is crucial to test if the magnitude and trend prior to the implementation are not significantly different compared to SSB. If the test fails, toiletries is not an adequate control group. This can be done with ITSA models with a control group. If the data set is at the household level, how was the specification for ITSA? Was it adapted to an ITSA? It is unclear as it seems that they are treating the data as time series given the autocorrelation test. I suggest to include the specification and provide a reference for ITSA models.

- We thank the reviewers for the suggestion about including details of the control (reference) group. We have added the following sentence to the manuscript (line 155): “Toiletries were selected as a suitable control group because purchasing is unlikely to be influenced by seasonality or confounders such as socioeconomic position.” The ITS of toiletries can be visualised in the graphs shown in Figures S1 of the supplement Text S3. The ITS on toiletries was not significant for changes in trend or step and this was built into our combined model and provides evidential proof that toiletries were an appropriate reference group. The dataset was not a household panel but a line list of purchases with demographical and household data linked as metadata.

Why are they modeling only sugar purchases and not also volume purchased? Did all brands reformulated?

- This was because the SDIL was not a sales tax based on volume sold. Rather, it was a two-tiered tax based on sugar density of drinks.

Is Kantar data nationally representative? What do you mean by "proprietary weight?
Why using an area level variable for socioeconomic status (SES) instead of household SES? How many missing data were in the data set? More than 10%? I suggest to provide results with and without the missing data.

- Please see response to the previous reviewer regarding additional detail on the Kantar dataset, weighting and specification of the interrupted time series model.
- During the study, we spend considerable time examining the potential influence of missing data. The proportion of ‘missingness’ is not consistent across the time frame of the analysis. The missing data are – by coincidence - very much restricted to the pre-announcement phase of the study, which has profound effects on the counterfactual. Later in the study, the level of missingness was almost nil.
- The counterfactual acts as the baseline against which we measure effect size, and small deviations in the trend in the pre-announcement phase may lead to quite large deviations in the counterfactual with extrapolation over longer and longer time periods. We felt that it would not be sensible to do an analysis without imputation because of the changing levels of missing data over the pre-announcement period.

The data is at the household level, I suggest to model purchases of sugar by adult equivalent or per capita and to adjust for household composition. Also, there are time varying macroeconomic variables that are associated with purchases, this should be included also.

- We include adjustments in the model for December and January, two months when there are spikes in purchases of soft drinks, and we also account for mean temperature of each month. There is strong evidence that more soft drinks are purchased over the warmer months.

In the results section, table 1, it is unclear how the estimations were derived from the ITSA model and if they included changes in level and slope.

- All estimates were derived from changes in slope and step. Please see model specification, which has been added as a supplement Text S1.

Results for the third quintile are positive and unexpected. This should be addressed.

- We agree that the small increase in sugar purchasing for the third quintile were unexpected. A similar pattern was found in analyses of the SDIL and childhood obesity [6], where a reduction in population prevalence of obesity was found in Year 6 girls in the most deprived groups (IMD 1 and 2) and the least deprived group (IMD 5), but not in the intermediate deprivation groups (IMD 3 and 4). In the Discussion (starting line 384) we speculate on the sensitivity of the results to the counterfactual (as mentioned in the response to the previous Reviewer comment) and the possibility of variations in of out-of-home purchases across IMD groups, which is not captured in the Kantar data . Other reasons for the unusual pattern could include changing demographics of the population over the course of the study, which may have led to more noise, or perhaps differences in susceptibility to marketing or advertising. We have added this text to the Discussion (line 386)

The time period covers the COVID-19 pandemic, if as in many countries there were changes in consumption patterns, this should be discussed and included in the model.

- The ITS analysis was stopped short of the COVID-19 pandemic, finishing in November 2019

For the modeling exercise, what is the assumption for the duration of the intervention?

- The intervention is assumed to be sustained over the lifecourse. Please see the response to Reviewer 3 for further discussion of this assumption.

Any attachments provided with reviews can be seen via the following link:

**Other author amendments:**

- We corrected an error in the display of data in Table 3 – the totals were showing the value for the first quintile, rather than the total of all quintiles. The error was in the prodution of the table rather than in the modelling; the totals were already reported correctly in the associated text.

**References**

1.	Coleman PC, Dhaif F, Oyebode O. Food shortages, stockpiling and panic buying ahead of Brexit as reported by the British media: a mixed methods content analysis. BMC Public Health. 2022;22(1):206. doi: 10.1186/s12889-022-12548-8.

2.	Rogers NT, Pell D, Mytton OT, Penney TL, Briggs A, Cummins S, et al. Changes in soft drinks purchased by British households associated with the UK soft drinks industry levy: a controlled interrupted time series analysis. BMJ open. 2023;13(12):e077059.

3.	Ananthapavan J, Nguyen PK, Bowe SJ, Sacks G, Mantilla Herrera AM, Swinburn B, et al. Cost-effectiveness of community-based childhood obesity prevention interventions in Australia. Int J Obes. 2019;43(5):1102-12.

4.	Forster M, Veerman JL, Barendregt JJ, Vos T. Cost-effectiveness of diet and exercise interventions to reduce overweight and obesity. Int J Obes. 2011.

5.	Mytton OT, Boyland E, Adams J, Collins B, O’Connell M, Russell SJ, et al. The potential health impact of restricting less-healthy food and beverage advertising on UK television between 05.30 and 21.00 hours: A modelling study. PLoS Med. 2020;17(10):e1003212.

6.	Rogers NT, Cummins S, Forde H, Jones CP, Mytton O, Rutter H, et al. Associations between trajectories of obesity prevalence in English primary school children and the UK soft drinks industry levy: An interrupted time series analysis of surveillance data. PLoS Med. 2023;20(1):e1004160.
