# Supplementary material for: Impact of the UK soft drinks industry levy on health and health inequalities in children and adolescents in England: An interrupted time series analysis and population health modelling study
Source: PLoS Med. 2024 Mar 28;21(3):e1004371. doi: 10.1371/journal.pmed.1004371 (PMC11008889; doi:10.1371/journal.pmed.1004371)
Supplement: Attachment — Submitted filename: Response to reviewers.docx [file pmed.1004371.s005.docx]

**Impact of the UK Soft Drinks Industry Levy on health and health inequalities in children and adolescents in England: an interrupted time series analysis and population health modelling study**

Response to reviewers’ comments

February 2024

Please find below our response to both the editors and the reviewers. Comments from editors and reviewers are provided verbatim, and our response is provided in blue.

-----------------------------------------------------------

COMMENTS FROM THE ACADEMIC EDITOR

I think now it is much clearer, their methods, their controls, etc. I think they answered carefully all the comments. I liked the new appendix and the clarity of their analysis. However, I concur that a major revision is needed to address the statistical reviewer comments.

COMMENTS FROM THE EDITOR-IN-CHIEF

The authors must discuss the strength of the causal inference and provide details of how secular confounding was ruled out.

- Please see response to reviewers to see how we have amended our manuscript accordingly.

COMMENTS FROM THE EDITORS

GENERAL

Thank you for your detailed responses to previous editor and reviewer comments. Please see below for further comments including from the statistical reviewer which we require that you address in full.

Due to the concerns raised by the statistical reviewer and the potential impact on the overall message conveyed by your paper, we have invited a further major revision for you to address these.

When re-submitting your manuscript, please include a clean, untracked and unhighlighted version with all changes accepted as well as a tracked version.

- Our response below demonstrates how we have addressed the concerns of the reviewers. Our response is accompanied by a clean and tracked version of the manuscript.

DATA AVAILABILITY STATEMENT

Thank you for updating your statement. Please also include a URL for Kantar and a contact email address for data enquiries.

- Since initial submission of this manuscript Kantar have updated their policy about academic publications using Kantar datasets. They have now insisted that they should not be named in the manuscript. Therefore we have removed all specific reference to Kantar in the manuscript and Data Availability Statement.

AUTHOR SUMMARY

Thank you for including an author summary.

As detailed previously, the author summary should constitute a non-technical summary of your research which makes findings accessible to a wide audience including both scientists and non-scientists. The author summary should consist of 2-3 succinct bullet points under each of the headings. The text should be distinct from the abstract.

In the final bullet point of ‘What Do These Findings Mean?’, please describe the main limitations of the study in non-technical language.

Please revise for brevity and improved accessibility to the non-scientist – we suggest removing all statistical information and describing your data in simple language.

- We have removed the statistical information and revised the text to improve clarity for a lay audience.

METHODS and RESULTS

We agree with the statistical reviewer (please see below) that further explanation of the life table model should be included in the main text (as opposed to the supplementary files). Please include.

- The lifetable model explanation has been moved from the supplementary file to the main text. This included replacing Figure 1 with Figure S1 – these model diagrams were very similar, but the S1 version provided a better illustration of the lifetable model states.

Lines 278, 287 & 300 – detail an ‘error’ related to citation of your supplementary files, perhaps confusion within the citation manager? Please check carefully throughout and amend this list is not exhaustive.

- We believe this was due to differences between the labelling of the supplement files and references to these files in the manuscript. This has been corrected.

FIGURES

Figure 1 – please ensure that all abbreviations and symbols (including those for units of measurement) are clearly defined in a footnote.

- We have edited Figure 1, as described above, such that it no longer includes the abbreviations and symbols

ACKNOWLEDGEMENTS

Please remove this statement in light of there being ‘none’.

- This has been removed

SUPPORTING INFORMATION

Throughout, please cite and label your Supporting Information (including individual tables and figures within the documents) as outlined here: <https://eur03.safelinks.protection.outlook.com/?url=https%3A%2F%2Fjournals.plos.org%2Fplosmedicine%2Fs%2Fsupporting-information&data=05%7C02%7CNina.Rogers%40mrc-epid.cam.ac.uk%7C528650226c9b4b73f97108dc138dbe4b%7C49a50445bdfa4b79ade3547b4f3986e9%7C1%7C0%7C638406746714718590%7CUnknown%7CTWFpbGZsb3d8eyJWIjoiMC4wLjAwMDAiLCJQIjoiV2luMzIiLCJBTiI6Ik1haWwiLCJXVCI6Mn0%3D%7C3000%7C%7C%7C&sdata=ZTNw%2F8453dvlNveytedicQPAGXpBTc6vTwv9s%2B6UCNw%3D&reserved=0>

- The manuscript complies with these requirements

S2 text – please ensure that all abbreviations used in tables and figures are clearly defined in a footnote or the caption.

- Abbreviations have been defined in the captions

S2 text, S5 Fig – please clearly state the meaning of the lines over the bars for the reader.

- Clarification added to caption

S3 text - please amend formatting to figure labels S1 Fig as opposed to Figure S1.

- Amended as requested

COMMENTS FROM THE STATISTICAL REVIEWER:

Reviewer #3: Thank you to the authors for replying in detail to the comments from myself and the other reviewers. There are still several (fairly major) aspects of the paper that I think need to be addressed before publication can be considered.

1. Similarity of this paper to the Rogers et al paper

I am still not convinced that this paper is different enough in its current format from the Rogers et al paper to justify a standalone paper. I will leave this issue to the journal editor(s).

- The Rogers et al paper did not include any lifetable modelling to investigate the medium- to long-term implications for health inequalities in England. The research question for this paper is “Did the Soft Drink Industry Levy reduce health inequalities in England?” which can only be answered through a longitudinal modelling approach (e.g. our lifetable modelling). The ITSAs which are also reported on here were conducted to provide inputs to the lifetable model. It was not possible to use the models that were reported in the Rogers et al paper as they were not appropriately specified to be compatible with our lifetable model (the main reason for this being that the lifetable model uses a simulated population of England, whereas the ITSAs reported in Rogers et al were for the entire of the UK). Therefore, the ITSAs reported in this paper are secondary to the main results, which are produced by the lifetable model. For this reason, we think there is very minimal overlap between the two papers.

2. Missing Data

Given the responses to my comment regarding missing data and the comment from Reviewer 4, I think a complete should definitely be presented as part of a sensitivity analysis. The fact that the missing data is by coincidence very much restricted to the pre-announcement phase of the study and therefore has a significant effect on the counterfactual is not a good enough excuse for not including this sensitivity analysis.

- We considered the issue of mssing data and imputation extensively during the study, this included discussions with Kantar (the data collectors) and other researchers who had also worked with these data sets, as well as conducting analyses both with and without imputation.
- Below are tables showing the impacts of the SDIL on sugar both with imputation (Table 1 – the results included in the manuscript) and without imputation (Table 2).
- Our key concern around imputation was that in this specific data set there was a high level of missing IMD values at the beginning of the study period but missing data were much lower by the end of the study period.
- Without imputation, the effects on sugar purchasing are over-estimated because the the increase in non-missing data makes it look as though the level of sugar purchasing is actually increasing (which we know to be incorrect) and this makes the difference between observed and counterfactual inflate over time.
- With imputation, the effects of the missing data inflating the difference between counterfactual and observed data disappears, giving a more accurate (and conservative) estimate of the SDIL effect on sugar purchasing.
- This importance of imputation can also be visualised by comparing Figure 1 below (a graphic of the imputed results currently included in the supplement) and Figure 2 (the same results without the imputation)

Table 1 : changes in sugar from soft drinks using imputed data. These numbers are based on being scaled to the population of England (0.865 with each quintile representing 0.2)

| Table 1 -imputed | Absolute change compared to counterfactual g | Relative change compared to counterfactual % |
| --- | --- | --- |
| Q1 | -37.5 (-31.5, -43.5) | -11.7 (-13.6, -9.87) |
| Q2 | -29.2(-23.4, -35.0) | -8.64 (-10.4, -6.92) |
| Q3 | 7.98(13.7, 2.23) | 3.03 (0.85, 5.21) |
| Q4 | -3.76 (1.85, -9.37) | -1.39 (-3.45, 0.68) |
| Q5 | -10.9 (-5.72, -16.0) | -4.08 (-6.02, -2.15) |
| Q1-5 combined | -15.0 (-10.3, -19.7) | -5.15 (-6.76, -3.53) |

Table 2 : changes in sugar from soft drinks. Includes an overall total but missing data category not included. These numbers are based on being scaled to the population of England (0.865 with each quintile representing 0.2)

| Table 2 -using non-imputed data | Absolute change compared to counterfactual g | Relative change compared to counterfactual % |
| --- | --- | --- |
| Q1 | -114.6 (-107, -121.6) | -28.9 (-30.7, -27.2) |
| Q2 | -102.9 (-96.0, -109.9) | -25.0 (-26.7, -23.3) |
| Q3 | -60.2(-53.7, -66.6) | -18.1 (-20.1, -16.2) |
| Q4 | -58.5(-52.4, -64.7) | -17.9 (-19.8, -16.1) |
| Q5 | -59.1(-53.3, -64.9) | -18.8 (-20.7, -17.0) |
| Q1-5 with no missing IMD data included | -79.5(-73.9, -85.0) | -22.3 (-23.9, -20.8) |

Figure 1 Impact of the SDIL on purchased sugar (g/household/week) in soft drinks by IMD quintile (Q1 is most deprived – Q5 is least deprived) – **WITH IMPUTATION**.

Figure 2 Impact of the SDIL on purchased sugar (g/household/week) in soft drinks by IMD quintile (Q1 is most deprived – Q5 is least deprived) – **WITHOUT IMPUTATION**.

3. How the optimal ITSA model was chosen

I am still not clear how the optimal ITSA was chosen on the basis of the AIC. Please be explicit and state how many different models were run, and the autoregressive order (p), moving average order (q) and AIC of the final model. Include this information either in the main text or (more likely) in the supplementary materials.

- Autocorrelation in the time series was investigated visually using plots of autocorrelation and partial autocorrelation and statistically using Durbin–Watson tests; an autocorrelation-moving average (ARIMA) correlation structure was used, with the order (p) and moving average (q) parameters chosen to minimise the Akaike information criterion (AIC) in each model. Overall, ten different models were run including options for p = 0, 1, 2, 4, 8 and q = 0,1,2,4,8. Furthermore,second and third order polynomial terms (for trend) were tested but were not found to improve the precision of the model in any case. This explanation has been added to S1 Text, which gives details on the structure of the ITSA model.

4. Lifetable Modelling Approach

I am still not convinced by the life table modelling approach used in the paper given the complexity of obesity as a disease area. Please include further discussion of the limitations of this life table modelling approach in the context of obesity.

- We acknowledge that obesity is complex, particularly at the level of the individual. However, it is important to note that in this paper we are chiefly concerned with trends in BMI at the population level, where much of the individual-level complexity is cancelled out. This is illustrated by looking at trends in BMI distributions,^1^ which are smooth and predictable. This type of lifetable modelling has a long history of being used to model the long-term health effects of population changes in body weight.^2-10^

5. Combining QALY impact of BMI and Dental Caries in a "multiplicative way"

The measurement and valuation of utility values is one of my research interests, and I've personally never heard of utilities being combined in a "multiplicative way". Please further justify this method, including references to both theoretical and applied paper (if applicable).

- The reviewer is correct – in this model the utilities are not being combined multiplicatively. We have edited the ‘Quality of life’ section in S2Text to remove this phrasing.

6. Explanation of the life table model in the main text

I still think further explanation of the life table model is needed in the main text, not the supplementary file. This is a key part of the paper.

- This has been moved to the main text, as described above.

**References**

1.	Cobiac LJ, Scarborough P. Modelling future trajectories of obesity and body mass index in England. *PLoS ONE* 2021; **16**(6): e0252072.

2.	Forster M, Veerman JL, Barendregt JJ, Vos T. Cost-effectiveness of diet and exercise interventions to reduce overweight and obesity. *Int J Obes* 2011.

3.	Cleghorn C, Jones A, Freeman L, Wilson N. Updated Cost-effectiveness Modelling of a Behavioural Weight Loss Intervention Involving a Primary Care Provider. New Zealand: University of Otago, 2020.

4.	Cleghorn C, Wilson N, Nair N, et al. Health benefits and cost-effectiveness from promoting smartphone apps for weight loss: multistate life table modeling. *JMIR mHealth and uHealth* 2019; **7**(1): e11118.

5.	Ekwaru JP, Ohinmaa A, Tran BX, Setayeshgar S, Johnson JA, Veugelers PJ. Cost-effectiveness of a school-based health promotion program in Canada: A life-course modeling approach. *PLoS ONE* 2017; **12**(5): e0177848.

6.	Veerman J, Barendregt J, Forster M, Vos T. Cost-Effectiveness of Pharmacotherapy to Reduce Obesity. *PLoS ONE* 2011; **6**(10): e26051.

7.	Mytton OT, Boyland E, Adams J, et al. The potential health impact of restricting less-healthy food and beverage advertising on UK television between 05.30 and 21.00 hours: A modelling study. *PLoS Med* 2020; **17**(10): e1003212.

8.	Haby M, Vos T, Carter R, et al. A new approach to assessing the health benefit from obesity interventions in children and adolescents: the Assessing Cost-Effectiveness in obesity project. *Int J Obes* 2006; **30**: 1463-75.

9.	Brown V, Ananthapavan J, Sonntag D, Tan EJ, Hayes A, Moodie M. The potential for long‐term cost‐effectiveness of obesity prevention interventions in the early years of life. *Pediatric obesity* 2019; **14**(8): e12517.

10.	Gortmaker SL, Long MW, Resch SC, et al. Cost effectiveness of childhood obesity interventions: evidence and methods for CHOICES. *Am J Prev Med* 2015; **49**(1): 102-11.
